# Reproducibility and relative validity of a food frequency questionnaire to assess dietary intake of adults living in a Mediterranean country

**DOI:** 10.1371/journal.pone.0218541

**Published:** 2019-06-17

**Authors:** Carla Aoun, Reine Bou Daher, Nada El Osta, Tatiana Papazian, Lydia Rabbaa Khabbaz

**Affiliations:** 1 Department of Nutrition, Faculty of Pharmacy, Saint-Joseph University of Beirut, Beirut, Lebanon; 2 Laboratoire de Pharmacologie, Pharmacie Clinique et Contrôle de Qualité des Médicaments, Faculty of Pharmacy, Saint-Joseph University of Beirut, Beirut, Lebanon; 3 Department of Public Health, Faculty of Medicine, Saint-Joseph University, Beirut, Lebanon; 4 Department of Prosthodontics, Faculty of Dental Medicine, Saint-Joseph University, Beirut, Lebanon; 5 Department of Prosthetic Dentistry, Saint-Joseph University, Beirut, Lebanon; 6 Centre de Recherche en Odontologie Clinique, University of Clermont Auvergne, Clermont-Ferrand, France; University of Extremadura, SPAIN

## Abstract

**Objective:**

Food frequency questionnaires (FFQs) must be validated among the population of interest due to the specificities in dietary habits, culture and food in each country of the Mediterranean region. The objective of this study was to determine the relative validity and reproducibility of a 157 item semi-quantitative FFQ among Lebanese adult population.

**Material and methods:**

Dietary intake was assessed through dietary recalls, a FFQ with food items, and traditional recipes from the Mediterranean cuisine. Validity of the FFQ was measured by comparing the intake of calories, macro and micronutrients to the mean values derived from three dietary recalls (DR). Reproducibility of the FFQ was evaluated after repeating the same FFQ among the participant after a four-month period.

**Results:**

114 healthy adults aged between 18 and 60 years of which 52.6% are men participated in this study. 53 of these adults participated in the reproducibility study. Intra class correlation coefficient (ICC) between the two FFQ measurements ranged from 0.822 for sodium to 0.998 for energy indicating excellent reproducibility. The FFQ showed slightly higher intakes than the dietary recalls for most of the nutrients and foods reaching 2.1% for nutrients (polyunsaturated fatty acids) and 18% for food groups (olive oil). Correlation coefficients ranged between 0.783 (sodium) and 0.996 (carbs) for nutrients and between 0.906 (fish) and 1 (fruits and nuts) for food groups, with a significant p value (p = 0.038 for folate). Cross-classification of nutrients into quartiles showed that more than 81% of the participants were classified in the same quartile. Misclassifications were low for most nutrients with one to three persons misclassified at the extreme quartiles.

**Conclusion:**

The FFQ used in this research contained western and Mediterranean type of dishes and foods. Statistical analysis showed good reproducibility and validity of the tested tool in a heterogeneous sample of adults living in a Mediterranean country. It is a useful tool for future investigations and strategies promoting the comeback of this traditional diet.

## Introduction

The assessment of diet is extremely important to the understanding and management of chronic diseases such as diabetes, hypertension, obesity, cancer, cognitive decline and dementia. Elucidation of diet /disease relationships requires dietary assessment methods that adequately describe and quantify intake, minimize systematic errors and provide reasonably precise estimates of variability between individuals and/or groups [1].

Over the last few decades, the Mediterranean diet (MD) has been at the heart of many epidemiological studies providing strong connection between diet and health outcomes. The rising interest for this eating pattern is not a trend anymore [2]. Indeed, one of the core characteristics of the Mediterranean dietary pattern is its high fiber content primarily from plant foods (i.e., vegetables, fruit, legumes, whole grains, and nuts), which is clearly shown to be associated with health benefits [3].

Unfortunately, in many countries in the Mediterranean area, the food habits are shifting toward an animal-based diet instead of a plant-based one, highlighting the conversion from a traditional MD to a Westernized one. This shift is caused by major transitions in social life, living conditions, and food intake of locals [4], [5]. Dietary habits are specific to each population and country in the Mediterranean region and no assessment method can be universal. Usually, these habits are examined through the use of validated instruments and scores, such as food frequency questionnaires (FFQ) that are frequently adopted as an assessment tool in most epidemiological studies and are essential for estimating intervention effects, monitor changes over time, and identify groups at high-risk. They are non-invasive, simple, cheap, easy to administer and interpret [1] [6].

The main difference between MD and Western diet is the sources and proportion of dietary fats. In Mediterranean diets, fat makes up about 30 percent of total calories, with most of the fat coming from olive oil (monounsaturated fat), and the consumption of meat and dairy products (saturated fats) are minimal, the overall intake of saturated fat is low. In fact, saturated fat is typically less than 10 percent of total calories in Mediterranean diets. Whereas olive oil supplies monounsaturated fatty acid, the fish, nuts and whole grains supply omega-3 and omega-6 fatty acids. Thus, there is ample supply of the essential fatty acids [7]. On the other hand, Western diet is characterized by high intakes of red meat, processed meat, prepackaged food, butter, fried food, and high fat dairy products. The 2010 Dietary Guidelines for Americans reports that solid fats from both saturated and trans fats contribute around 19 percent of calories to the American diet.

Before being used, FFQs must be validated among the population of interest due to the specificities in dietary habits, culture and food in each country of the Mediterranean region [8]. Furthermore, FFQs must be validated in the sub-population of interest: i.e pregnant women, elderly, children or adolescents, since they may have specific habits, different from the adult general population. In the Middle East and North Africa (MENA) region, FFQs have been validated among adults in the Islamic Republic of Iran [9], United Arab Emirates, Kuwait [10], and Jordan [11]. However none of these FFQs can represent the dietary or culinary habits of the Lebanese population because in the Mediterranean region every country has its own food ingredients, traditions and culinary habits.

Lebanon is a small country on the Eastern shore of the Mediterranean Sea and is known for its rich traditional cuisine with a large variety of ingredients. The main components of this diet are cereals, legumes, vegetables, nuts, fish, olive oil, and fruits [12]. In 2011, the adherence of Lebanese population to the Mediterranean diet and the relationship with metabolic syndrome was studied using a 78-items qualitative FFQ describing only simple ingredients without mentioning composite dishes which have a major place in the Lebanese diet [13]. In 2016, two FFQs were validated in Lebanon, one among pregnant women [14] and another among children [15]. However, there is a need to adapt and validate an FFQ that can be used as a valid tool in the future to accurately assess the nutritional intakes of the general population living in Lebanon and in surrounding countries presenting similar dietary habits.

The food items mentioned in this FFQ are typical and representative of the Lebanese diet and consequently the aim of this study is to assess the relative validity and reproducibility of a 157-items semi-quantitative FFQ containing Middle Eastern and Mediterranean food.

## Materials and methods

### 2.1. Study design

This cross-sectional study is a part of a larger study evaluating the adherence of the Lebanese population to the traditional Mediterranean diet. And in order to use this FFQ in a larger national cross-sectional study it must be validated on a sample of Lebanese adults. Participants completed two FFQs and three 24 hours dietary recalls (24h DR).

### 2.2. Study population

The total number of participants was 114 healthy adults of both genders aged between 18 and 60 years. There are no specific guidelines to determine the sample power for testing the performance of FFQ. But the number of participants was arbitrarily set at 114 taking into account the sample size used in previous studies [14] [16] [1].

We followed a random household sampling procedure involving door-to-door recruitment and face-to-face contact. We chose randomly a number of houses proportional to the population density of the area. Individuals were presented with the study information sheet, and were screened using a questionnaire on medical history. Prior to data collection, participants consented to the study and the procedure.

The participants were recruited from all Lebanese geographic districts including the capital Beirut and regions in Mount Lebanon, North, South and Bekaa. The following sub-groups were excluded: pregnant and breastfeeding women, women who gave birth less than 6 months ago, those suffering from a chronic disease, an acute medical condition the week before the inclusion and those on continuous treatment. The study was conducted from March 2017 to December 2017. The study protocol was approved by the Institutional Review Board of Saint-Joseph University Beirut and its Ethic committee (USJ-2016-62). And it is to be mentioned that all subjects gave their written consent before they were included in the study.

### 2.3. Methodology

Pairing of interviewee-to-interviewer remained constant throughout the study to maintain consistency in data collection and analysis of dietary intake [17] [18] [19].

The study was divided into three parts:

In the first part, participants were required to give detailed sociodemographic information including age, health status, socio-economic situation through household crowding index, educational and work level [20]. The second part concerned the anthropometric measurements like waist circumference, Body Mass Index (BMI) and Waist to Hip ratio (WHR) [21]. The third part was related to the dietary intakes assessed based on the 157 items semi quantitative FFQ.

The FFQ was developed and tested in a pilot study by a panel composed of researchers and nutrition instructors developed the questionnaire. It is divided into 12 food groups depending on the nutritional composition of the items, and in each group we chose the items that are most consumed and representative of the Lebanese cuisine: cereals and bread, rice and grains, milk and dairy products, fruits and juices, vegetables, meat and alternatives, spices and nuts, sweets, bakeries, salty snacks, oils and fats and finally alcohol [14]. The overall intake refers to the previous year, and frequency of food consumption was divided into four open-frequency categories of response to facilitate the estimation for the participant and avoid underreporting: times per day, week, month, and never [14]. The FFQ is provided as a supplement material. Participants were assisted in their estimation of portion sizes with plastic food models and local food photos. This type of FFQ is very common and has a high assessment value evaluating long term nutrition intake especially that people suffering from any condition that affects the dietary intakes were excluded from the study.

The same interviewer filled a 24h DR for 3 different separated days within the following week via phone calls, including a non-working day. Participants described the previous day consumption qualitatively and quantitatively using household measures, to help the dietitian estimate total intake. No previous warning was given to the participants on the day on which they would be called [22]. Finally, a second FFQ was administered four months later in a face-to-face interview conducted by the same researcher, among almost half the participants. The sample size selected to test reproducibility was based on what was recommended by Cade et al. and Willet et al. recommending the inclusion of at least 30% of the initial sample randomly chosen [23] [1]. Each participant had the same interviewer to reduce possible bias.

**Fig 1:**
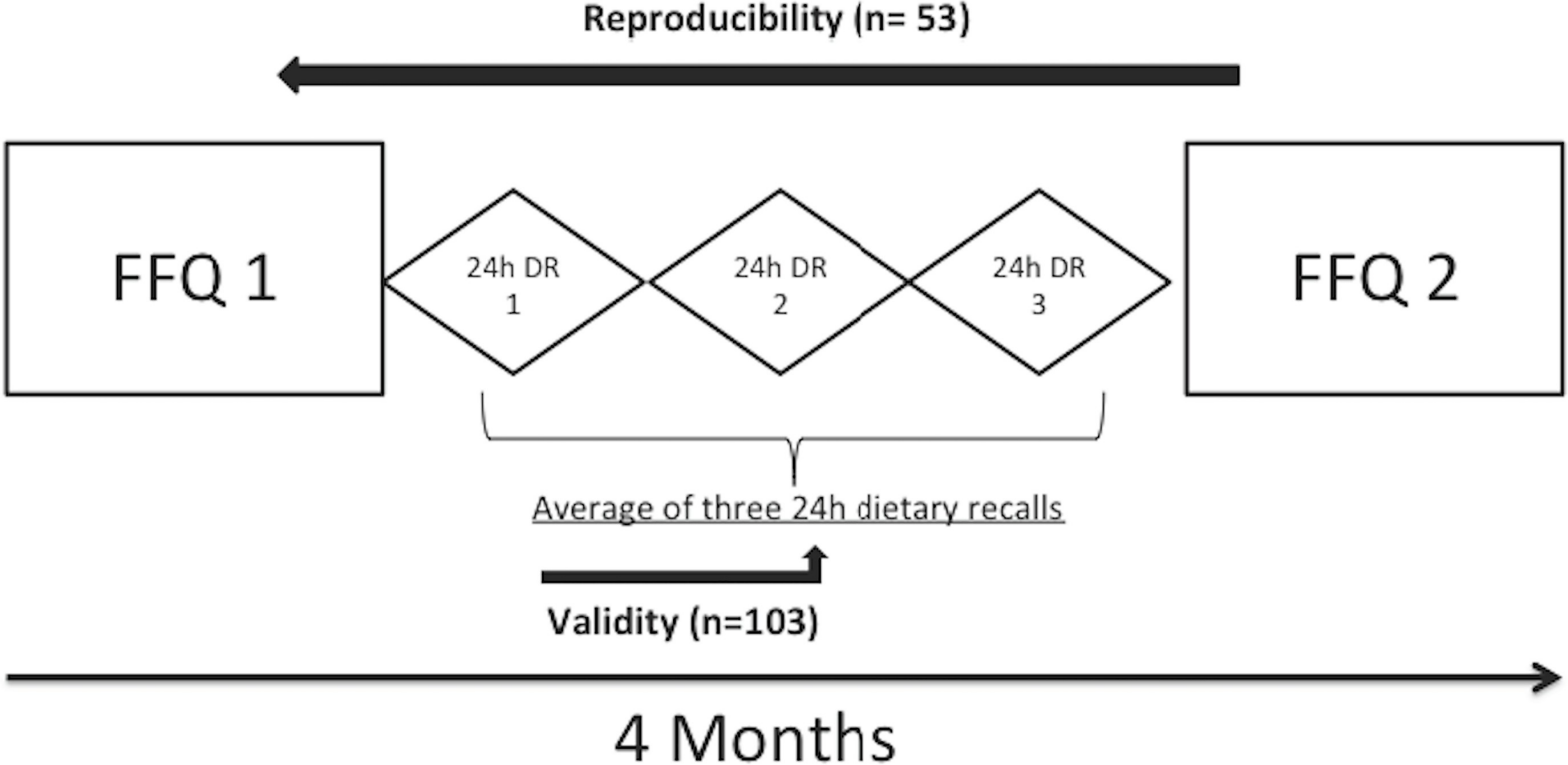
The design of the validation and reproducibility study among Lebanese adults.

For reproducibility, the participants completed a second FFQ four months later. Validity of the FFQ was assessed against the 24h DR as described elsewhere [24] [25] [26]. Three 24h DRs were completed during the week after the first FFQ. The three days were predetermined by the investigator, they were non-consecutive and included one non-working day like described in previous similar studies [15][27]. (Fig 1). Cade et al. in a systematic review found that most validation studies used the 24-h recall questionnaires as reference method against FFQs [23].

### 2.4. Nutrient intake calculation

To process all information provided by the FFQ, frequencies were converted into daily intake in grams. Then, to analyze the FFQ and DRs recalls and to estimate the energy and nutrients intakes we used the Nutrilog 2.3 software. For international food items, nutrients values were obtained from the United States Department of Agriculture (USDA) database and the French food composition table CIQUAL [28] [29]. For Lebanese food recipes, the database of the American University of Beirut was used [30].

Missing or incomplete DRs as well as participants having intakes below 500 kcal and above 3500 kcal per day were excluded [31] [18]. We started with 125 participants, out of which six had under or over reported energy intakes and five found it exhaustive to complete three DR. The final pool included 114 participants. Descriptive analyses were completed for energy intake, 7 food groups representative of the Mediterranean Diet (Vegetables, legumes, fruits and nuts, cereals, meat, fish and olive oil) and 31 nutrients measured by both FFQs and the three 24h DRs.

#### 2.4.1. Relative validity of the food frequency questionnaire

Means and standard deviations were calculated for energy and nutrient intakes for the FFQ and the three 24h DRs. Kolmogorov-Smirnov test was used to evaluate the normality of distribution. Almost all the food groups and nutrients followed a normal distribution. Non-parametric tests were conducted when the variables did not show a normal distribution. Paired Student's test or its non-parametric equivalent Wilcoxon test was used to compare variables from FFQ and Recalls. Also, Bland Altman Plots were carried out to assess agreement between the two tools for total energy intake, food groups, macronutrients and micronutrients; the differences of the two measurements were plotted against the mean of the two methods [32]. Finally, the kappa statistic (*K*) was calculated by comparing intake quartiles for each nutrient in the FFQ and the 24h DRs. According to Landis et al. the following values were used to evaluate the level of agreement between the two methods: ≥ 0.80 indicates very good agreement, *K* = 0.61–0.80 indicates good agreement, *K* = 0.41–0.60 indicates moderate agreement, *K* = 0.21–0.40 indicates poor agreement, and *K* ≤ 0.20 indicates very poor agreement [33].

#### 2.4.2. Reproducibilty of the food frequency questionnaire

Means and standard deviations were used to assess nutrient intakes in both FFQs (FFQ1 and FFQ2). To measure the FFQ reproducibility, we calculated intra-class correlation coefficients between values obtained in the two FFQs. To evaluate the reproducibility we used the following classification: Poor reliability (<0.5), moderate reliability (0.5 to 0.75), good reliability (0.75 to 0.90), and excellent reliability (>0.9). All statistical analyses were performed using the SPSS statistical software (SPSS for windows, version 24.0, USA). A p value < 0.05 was considered as significant.

## Results

### 3.1. Sociodemographic and anthropometric characteristics of the population

The mean age of the participants was 33 years (±12.8SD) equally distributed between men and women (52.6% men, 47.4% women). The age distribution is represented in Table 1 and showed that The percentage of young people in Lebanon is higher than older one. All age groups are well represented equally.

**Table 1:** Age distribution of the sample.

Age (years)	Frequency	Percentage
18–25	38	33.3%
26–35	37	32.5%
36–50	21	18.4%
51–60	18	15.8%

The mean crowding index was 0.93 (±0.42 SD). 39.5% of the participants had a normal Body Mass Index (BMI), 32.5% were overweight and 20.2% obese according to the criteria of World Health Organisation [34]. 32.5% of the participants were smokers.

### 3.2. Reproducibility of the food-frequency questionnaire

53 Lebanese adults participated in the reproducibility study. The protocol was to administrate the same FFQ twice under the same conditions (same interviewer) four months apart. The mean daily intakes of energy, nutrients and food groups obtained from the two questionnaires are presented in **Table 2**. Intra class correlation between the two FFQ measurements ranged from 0.822 (sodium) to 0.996 (carbs) for nutrients; and 0.933 (olive oil) to 0.996 (vegetables) for food groups indicating excellent reproducibility (p ≤ 0.05).

**Table 2:** Reproducibility study: Mean daily energy and nutrient intakes and the intra-class coefficient for the comparison between FFQ1 and FFQ2 in Lebanese adults.

	FFQ1 Mean ± SD	FFQ2 Mean ± SD	ICC (with 95% CI)
Energy (Kcal)	2096 ± 543	2084 ± 539	0.998 [0.996–0.999]
Carbs (g)	232 ± 70	229 ± 69	0.996 [0.993–0.998]
Carbs (%)	45 ± 8	45 ± 8	0.991 [0.985–0.995]
Proteins (g)	86 ± 30	86 ± 30	0.994 [0.990–0.997]
Proteins (%)	16 ± 3	16 ± 3	0.983 [0.970–0.990]
Fat (g)	84 ± 29	83 ± 29	0.995 [0.991–0.997]
Fat (%)	36 ± 6	36 ± 6	0.984 [0.973–0.991]
Sugars (g)	63 ± 35	63 ± 35	0.995 [0.992–0.997]
Fibers (g)	16 ± 5	16 ± 5	0.952 [0.918–0.973]
Iron (mg)	12 ± 5	12 ± 4	0.982 [0.969–0.990]
Calcium (mg)	913 ± 598	915 ± 602	0.996 [0.993–0.998]
Sodium (mg)	4440 ± 657	4343 ± 835	0.822 [0.699–0.897]
Vitamin D (μg)	10 ± 5	11 ± 5	0.983 [0.971–0.990]
Folic acid (μg)	210 ± 98	205 ± 88	0.978 [0.962–0.987]
Vitamin A (μg)	375 ± 356	327 ± 260	0.834 [0.712–0.904]
Magnesium (mg)	216 ± 82	208 ± 76	0.968 [0.945–0.982]
Zinc (mg)	8 ± 4	8 ± 4	0.993 [0.988–0.996]
Potassium (mg)	2527 ± 1111	2474 ± 1083	0.993 [0.987–0.996]
Saturated fatty acids (g)	23 ± 12	24 ± 13	0.989 [0.981–0.994]
Monounsaturated Fatty acids (g)	26 ± 10	26 ± 10	0.978 [0.961–0.987]
Polyunsaturated Fatty acids (g)	10 ± 4	9 ± 4	0.949 [0.912–0.971]
Alcohol (g)	7.9 ± 14.323	8.1 ± 14.303	0.996 [0.993–0.998]
Alcohol %	2.8 ± 5.008	2.9 ± 4.996	0.996 [0.994–0.998]
Vitamin E (mg)	7 ± 5	7 ± 5	0.981 [0.966–0.989]
Vitamin C (mg)	69 ± 55	62 ± 37	0.835 [0.714–0.905]
Vitamin B1 (mg)	1 ± 0.4	1 ± 0.4	0.940 [0.897–0.965]
Vitamin B12 (mg)	4 ± 2.8	4 ± 2.5	0.983 [0.971–0.990]
Manganese (mg)	1.9 ± 0.7	1.8 ± 0.6	0.920 [0.862–0.953]
Selenium (μg)	68.3 ± 29.0	71.6 ± 32.7	0.966 [0.941–0.980]
Vegetables (g/day)	156.94 ± 110.32	165.02 ± 112.59	0.996 [0.995–0.998]
Legumes (g/day)	37.55 ± 30.64	36.37 ± 31.71	0.982 [0.974–0.988]
Fruits and nuts (g/day)	202.52 ± 125.45	204.32 ± 125.56	0.995 [0.993–0.997]
Cereals (g/day)	172.96 ± 94.35	172.91 ± 95.67	0.996 [0.994–0.997]
Meats (g/day)	91.62 ± 54.73	92.50 ± 54.10	0.996 [0.994–0.997]
Fish (g/day)	33.89 ± 33.63	34.81 ± 33.64	0.994 [0.991–0.996]
Olive oil (g/day)	8.34 ± 6.47	8.20 ± 6.25	0.933 [0.904–0.953]

### 3.3. Relative validity of the food-frequency questionnaire

To assess the validity of the FFQ, we chose to compare the intake of energy, macro and micronutrients to three 24h DRs [35] [14] [36] [37] [38] [39]. Descriptive statistics and Spearman and Pearsons correlations between nutrient intakes derived from the FFQs and the three 24h DRs are presented in **Table 3**. The FFQ showed slightly higher intakes than the DRs for most of the nutrients and food groups reaching 2.1%(polyunsaturated fatty acids) for nutrients, and 18.1%(olive oil) for food groups. Correlation coefficients ranged between 0.783 (Sodium) and 0.989(carbs) for nutrients and from 0.976 (olive oil) to 1 (fruits and nuts) for food groups with a highly significant p value indicating a good correlation. Cross-classification analysis showed that more than 81% of the participants were classified in the same quartile (93.2% for protein intake, 95.2% for fat intake, 87.4% for vitamin A, 93% for meat consumption and 96.5% for vegetables consumption). The proportion of subjects classified in the same quartile ranged from 81.1% (vitamin D) to 99% (fat) for nutrients and from 92.1%(fish) to 99.1% (fruits and nuts) for food groups. The proportion of subjects classified in the extreme quartile was less than 3%, with the highest value obtained for vitamin D and Polyunsaturated fatty acids (2.9%). Kappa values ranged between 0.71 (vitamin B1) and 0.98 (folic acid) for nutrients and from 0.74 (olive oil) to 0.98 (fruits and nuts) for food groups indicating good and very good agreement.

**Table 3:** Validation study: FFQ and three 24h DRs. Comparison of mean daily intake of energy and nutrients.

	24h DR Mean ± SD	FFQ Mean ± SD	p-value ^a^	Relative difference (%) ^b^	Correlation coefficient^c^	
					Unadjusted	Energy Adjusted
Energy (Kcal)	2004.971 ± 549.47	2011.18 ± 553.64	0.098	0.3%	0.998	0.998
Carbs (g)	224.38 ± 71.25	225.46 ± 71.61	0.046	0.5%	0.996	0.989
Carbs (%)	45.07 ± 7.83	45.16 ± 7.79	0.275	0.2%	0.995	0.995
Proteins (g)	81.41 ± 28.01	81.22 ± 28.64	0.001	-0.2%	0.996	0.989
Proteins (%)	16.22 ± 3.09	16.12 ± 3.22	0.011	-0.6%	0.987	0.986
Fats (g)	80.27 ± 28.05	80.23 ± 27.93	0.877	0.0%	0.996	0.986
Fats (%)	36.09 ± 6.46	35.96 ± 6.38	0.082	-0.4%	0.993	0.993
Sugars (g)	60.32 ± 29.04	59.92 ± 29.32	0.055	-0.7%	0.993	0.988
Fibers (g)	15.59 ± 5.29	15.70 ± 5.48	0.397	0.7%	0.970	0.950
Iron (mg)	11.96 ± 4.87	11.81 ± 4.88	0.073	-1.3%	0.967	0.976
Sodium (mg)	4412.26 ± 767.07	4308.24 ± 756.53	0.010	-2.4%	0.909	0.783
Folate (μg)	204.57 ± 95.66	202.72 ± 94.59	0.038	-0.9%	0.978	0.970
Magnesium (mg)	202.05 ± 74.18	205.15 ± 75.93	0.075	1.5%	0.973	0.937
Saturated fatty acids (g)	22.78 ± 11.26	22.51 ± 11.25	0.320	-1.2%	0.969	0.934
Monounsaturated fatty acids (g)	24.15 ± 9.35	24.52 ± 9.47	0.126	1.5%	0.967	0.927
Polyunsaturated Fatty acids (g)	9.26 ± 4.52	9.46 ± 4.53	0.081	2.1%	0.969	0.958
Alcohol (g)	6.93 ± 11.77	7.46 ± 12.59	0.007	7.6%	0.990	0.990
Alcohol (%)	2.46 ± 4.03	2.63 ± 4.31	0.018	7.2%	0.986	0.986
Vitamin C (mg)	62.88 ± 42.57	62.22 ± 43.57	0.348	-1.1%	0.987	0.982
Vitamin B12 (mg)	3.83 ± 2.28	3.76 ± 2.31	0.289	-1.8%	0.973	0.945
Phosphorus (mg)	1209.86 ± 462.44	1226.45 ± 472.87	0.003	1.4%	0.993	0.981
Copper (mg)	0.97 ± 0.36	0.94 ± 0.34	0.001	-2.7%	0.904	0.797
Vegetables (g/day)	152.91±107.14	160.98±111.36	<0.001	5.0%	0.998	
Legumes (g/day)	34.08±30.07	36.96 ± 31.04	<0.001	8.0%	0.998	
Fruits and nuts (g/day)	198.43±124.20	203.42±125.4	<0.001	2.5%	1.000	
Cereals (g/day)	160.73±87.77	172.93 ± 94.90	<0.001	6.9%	0.997	
Meats (g/day)	88.42±53.58	92.06±54.36	<0.001	3.9%	0.996	
Fish (g/day)	29.56±28.75	34.35±33.59	<0.001	13.9%	0.906	
Olive oil (g/day)	6.77±5.09	8.27±6.26	<0.001	18.1%	0.976	

a Paired Student t tests or Wilcoxon signed rank test were used to compare values obtained from both FFQ and 3-24h DRs

b Relative difference = [(FFQ– 24h DR)/24h DR]*100.

c Pearson or Spearman correlation coefficient were used to assess the correlation between variables with a p-value < 0.05 considered as significant

### 3.4. Bland-Altman analyses

**Fig 2:**
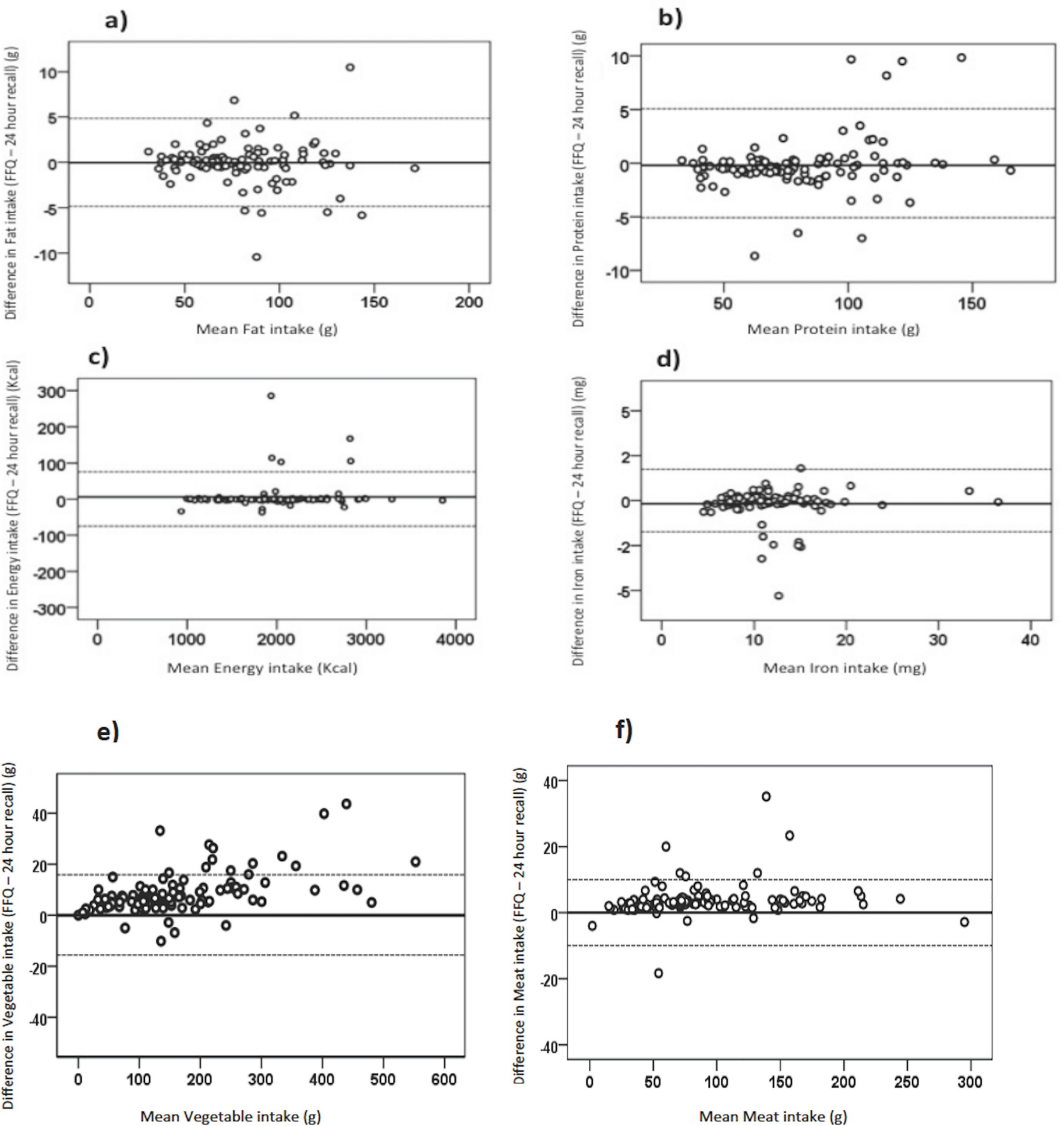
Bland-Altman plot representing the agreement between the average of nutrient intakes measured from the FFQ and the 24f DRs. The solid line represents the average difference between the two methods used (FFQ and 24h DRs), while the dashed line represents the distance between the limits of agreement (± 2SD). (a) Bland-Altman analysis for fat intake; (b) Bland-Altman analysis for protein intake; (c) Bland-Altman analysis for energy intake; (d) Bland-Altman analysis for iron intake; (e) Bland-Altman analysis for vegetable intake; (f) Bland-Altman analysis for meat intake.

Bland-Altman plot is a graphical representation that consists of studying the agreement of nutrient measured from the FFQ and the dietary recalls, some of which are shown in Fig 2. The solid line represents the average difference between the two methods used (FFQ and dietary recalls), while the hatched line represents the distance between the mean of the difference ± 2 standard deviations. This is the limit of the "Limit of agreement" agreement.

The limits of agreement were adequate and varied between negative and positive values, and showed that nutritional intakes were both over- and underestimated by FFQ compared to the 24h DRs. Fig 2 represents the Bland-Altman analysis of Fat, protein, energy and iron intake showing a homogeneous dispersion above and below zero in most plots. The Bland–Altman method is the most relevant to evaluate absolute validity by estimating mean agreement and the limits of agreement. Mean agreement indicates the mean of the difference between the assessment tools used to determine food intake. This value was almost the same with the two assessment tools (the FFQ and the 24h DR), which indicate a good degree of absolute validity. For nutrient intakes, the Bland-Altman analysis showed a percentage of agreement ranging from 93% for carbs to 98% for sodium. Regarding food groups the percentage of agreement ranged between 80% for legumes and 94% for meats.

## Discussion

Other similar studies previously published among the general Lebanese adult population were conducted without validating the FFQ before using it among the participants [40].

Assessing dietary habits and nutritional intakes of populations is of great interest to researchers. However, differences in cultures and dietary habits between populations make it necessary to create culturally specific tools to evaluate food intakes. This is why we chose to conduct the first FFQ validation study of a culturally specific FFQ among Lebanese adults from all geographic districts. One previous study validated the same FFQ on a population of Lebanese pregnant women and another FFQ was validated on children [14] [15].

Our FFQ was carefully designed to merge food items reflecting the traditional Mediterranean diet and at the same time items representing a Westernized dietary pattern due to the nutritional transition affecting us [41][42]. Our methodological strategy was to combine several statistical methods, in order to assess the relative validity and reproducibility of the developed FFQ.

Knowing that our FFQ represented the intakes over the past year, the four-months laps between the two FFQs was considered reasonable. According to Willet, in conducting reproducibility study, it is unrealistic to administer the questionnaire at a very short interval, a few days or weeks, because subjects may simply tend to remember their previous responses. If a questionnaire refers to intake over the past year, administrations a few months apart should largely reflect variations associated with completing the questionnaire [18].

The FFQ administered four months apart showed reasonably high reproducibility with correlation coefficients ranging from 0.822 (Sodium) to 0.998 (Energy) for most nutrients and food groups, which may be considered as a very good correlation. These values were higher than reported by Morel et al. in 2018 in a French-Canadian study ranging from 0.56 to 0.88 [43] but similar to those published by Fallaise et al. in 2014 that ranged from 0.65 to 0.9. [44].

In Lebanon, food is constantly consumed throughout the seasons because of its continuous availability and therefore, nutritional intake doesn’t undergo drastic variations between seasons, which may explain the high correlation seen between the two FFQs. In addition, the same interviewer, either a professional dietitian or a trained researcher, collected data from the same patient which also contributed to lessen the variability [16]. Finally, holiday seasons were avoided during the collection of data because they don’t capture usual dietary intake, which contributed also to the high correlation.

For the validity study of the FFQ, according to Ma et al, three 24h DR appear optimal for estimating dietary intakes [45]. The correlation coefficients found between nutrients derived from FFQs and the three 24h DR ranged between 0.783 (Sodium) and 0.998 (Vegetables). These values were relatively higher than those reported by Cade et al. and Willet et al. (0.5 to 0.7) [23] [1]. These high values obtained for correlation coefficients (> 0.5) for most of the nutrients could be explained by the complete and detailed food list in the FFQ, the method of administration (same interviewer conducted the study, type of question, the easy facilitated estimation of portions) and the short period of time between the first FFQ and the 3 DRs. According to Tsubono et al, correlation coefficients tended to be lower when FFQ were repeated after a long time interval (6 months to 1 year) compared with a shorter time interval (1 to 6 months), which could be explained by the variation in dietary habits due to longer time laps [46]. In addition, the exclusions of participants under or over estimating their total energy intakes, or those suffering from any condition that could affect the nutritional intake or status, lead to stronger correlations.

Regarding categorization into intake levels, the quartile notation in this study was good and comparable to those of previously reported by Zaragoza et al. showing that more than 84% of the subjects were classified in the same quartile [47]. Our finding that the FFQ slightly overestimates most of the nutrient intakes are consistent with those described by other researchers reaching 6.9% for cereal and 18.1% for olive oil intake [11], [48].

Finally, the Bland Altman analyses were used to illustrate the agreement between the FFQ and the 24h DR by comparing the differences between the tools against the mean of the two tools. When the mean of differences is closer to zero it indicates a narrower agreement interval and therefore a better agreement between the two tools. Therefore, this FFQ showed a very good validity compared to the DRs.

### 4.1. Strengths/limitations

According to some recent investigations, there is an escalating debate over the value and validity of self-reported dietary intake as estimated by FFQs and other forms of memory-based dietary assessment methods [49]. They are considered to be pseudo-scientific and non-reliable methods to study the diet-disease relationship and some scientists are pushing towards more rigorous scientific methods like RCTs to examine the effect of diet on chronic diseases[50]. Even though the limitation of these assessment methods is acknowledged, FFQs remain until nowadays the most used dietary assessment method to study dietary patterns and population behaviors.

Validating the FFQ on a wider sample would be interesting, since this study excluded many categories of population (pregnant and breastfeeding women and those suffering from chronic diseases); these exclusions might limit the use of this FFQ to the general population. In addition, despite the fact that the FFQ was validated against multiple DRs, which is commonly used as a method for validation, further validation with biomarkers would best support our results [16] [1]. Another limitation in our study is that we conducted the three DRs during the week following the first FFQ; it would have been more representative if spread throughout the year. Due to the limited time frame and budget constraints of our study this was not possible.

Another potential source of error common to self-reported dietary methods is in the estimation of nutrients with use of food composition tables. The nutritional composition of a food varies with season, location of production, growing conditions, storage, processing, and cooking techniques, and many of these factors are unaccounted for in food composition tables. Estimating nutrient composition from food intake data is a challenge, and it is crucial that we continue improving the accuracy of food composition databases [51].

On the other hand, the main strength of this study is that the same trained interviewer conducted the study for the patient, which has probably minimized the variability in estimating the portions and the intakes. Also, our list of food was adapted to the modern lifestyle of a typical Mediterranean citizen and therefore this FFQ represents in the best possible way the dietary pattern of this population. The statistical methodology applied in this paper is a further strength. Even though Lombard et al. in a recent review considered one to three statistical tests to be acceptable for validation studies [52], we combined many statistical approaches to assess the reproducibility and validity of this tool.

Time constraints, financial and participant burden are critical research issues. That’s why future investigations in this field should focus on developing online questionnaires to assess nutritional intakes. The use of computerized systems in epidemiology can be beneficial by allowing faster responses, reducing the bias of incomplete data via automated controls and making the estimation of food portions easier through illustrated models [53].

## Conclusions

The Mediterranean dietary pattern is very well documented and has shown high nutritional quality and beneficial health outcomes. Many health promotion strategies nowadays are drawing attention to the Mediterranean diet and these initiatives need a valid tool to assess the nutritional intakes of a population.

The objective of this study was to adapt a 157-item semi-quantitative FFQ already developed and validated on a population of Lebanese pregnant women, to assess its validity and reproducibility among the general adult Lebanese population [14]. The FFQ validated in this study showed good reproducibility and relative validity compared to three 24h DRs in a heterogeneous sample of adults living in a Mediterranean country.

In conclusion, we offer a valid FFQ that could be helpful in estimating food groups and nutrient intakes and useful for the investigation of dietary risk factors and Non Communicable Diseases. This FFQ is also suitable for clinical trials including nutritional interventions promoting the Mediterranean diet. Future works could focus on developing and validating a computerized version of this FFQ in order to limit its administration time.

## Supporting information

S1 File FFQ in Arabic. (DOCX)

S2 File FFQ in English. (DOCX)
