# Supplementary material for: Reproducibility and relative validity of a food frequency questionnaire to assess dietary intake of adults living in a Mediterranean country
Source: PLoS One. 2019 Jun 17;14(6):e0218541. doi: 10.1371/journal.pone.0218541 (PMC6576765; doi:10.1371/journal.pone.0218541)
Supplement: S1 File — (DOCX) [file pone.0218541.s001.docx]

**القسم الثاني: استمارة غذاء (أسئلة حول الإستهلاك اليومي للأطعمة**)

|  | **الطعام** | **الحصة** | **عدد الحصص** | **عدد مرّات الإستهلاك** | | | | |
| --- | --- | --- | --- | --- | --- | --- | --- | --- |
|  |  |  |  | **في اليوم** | **في الأسبوع** | **في الشهر** | **أبدًا** | |
| **A** | **الخبز والحبوب** | | | | | | | |
| 1 | الخبز العربي (منها السندويشات( | **¼ رغيف كبير**  **½ رغيف وسط** |  |  |  |  |  | |
| 2 | الخبز المرقوق / خبز التنّور | **1 تنور**  **½ مرقوق** |  |  |  |  |  | |
| 3 | خبز الهمبرغر | **1 قطعة** |  |  |  |  |  | |
| 4 | خبز فرنجي (باغيت) | **30 غ**  **أو حجم صغير** |  |  |  |  |  | |
| 5 | خبز بالحليب (Pain au lait) | **1 قطعة** |  |  |  |  |  | |
| 6 | توست، كراكوت | **3 قطع** |  |  |  |  |  | |
| 7 | بان دومي | **قطعتين** |  |  |  |  |  | |
| 8 | كعك | **8 قطع مدوّرة**  **3 أصابع وسط**  **1 إصبع كبير** |  |  |  |  |  | |
|  | ما هو نوع هذه المأكولات؟ | € أبيض € أسمر (قمحة كاملة) € غير ذلك: .................... | | | | | | |
| 9 | كورن فلايكس عادي | **½ كوب** |  |  |  |  |  | |
| 10 | كورن فلايكس غنيّ بالألياف | **¾ كوب** |  |  |  |  |  | |
| 11 | لوح حبوب (Barre de Céréales) | **30 غ**  **أو 1 قطعة** |  |  |  |  |  | |
| 12 | شوفان | **½ كوب** |  |  |  |  |  | |
| **B** | **البطاطا والمعكرونة والأرز والحبوب** | | | | | | | |
| 13 | أرز مطبوخ | **كوب**  **(15 معلقة كبيرة)** |  |  |  |  |  | |
| 14 | محاشي | **12 ورق عنب**  **6 ملفوف 3 كوسى** |  |  |  |  |  | |
| 15 | برغل مطبوخ / فريكة مطبوخة / مغربية | **كوب**  **(10 ملاعق كبيرة)** |  |  |  |  |  | |
| 16 | كبّة | **1 قطعة وسط**  **أو قرصين** |  |  |  |  |  | |
| 17 | معكرونة أو باستا مطبوخة | **كوب** |  |  |  |  |  | |
| 18 | بطاطا (منها المهروسة أو بوريه) | **١ قطعة وسط**  **أو كوب** |  |  |  |  |  | |
| 19 | بطاطا مقلية | **10 -12 قطعة** |  |  |  |  |  | |
| 20 | كستناء | **4 – 5 قطع** |  |  |  |  |  | |
|  | **الطعام** | **الحصة** | **عدد الحصص** | **عدد مرّات الإستهلاك** | | | | |
|  |  |  |  | **في اليوم** | **في الأسبوع** | **في الشهر** | **أبدًا** | |
| 21 | كينوة | **5 ملاعق كبيرة** |  |  |  |  |  | |
| 22 | بازيلا | **½ كوب**  **(5 ملاعق كبيرة)** |  |  |  |  |  | |
| 23 | ذرة | **½ كوب**  **(5 ملاعق كبيرة)** |  |  |  |  |  | |
| 24 | فول، فاصوليا | **كوب**  **(10 ملاعق كبيرة)** |  |  |  |  |  | |
| 25 | عدس | **كوب**  **(10 ملاعق كبيرة)** |  |  |  |  |  | |
| 26 | حمص | **كوب**  **(10 ملاعق كبيرة)** |  |  |  |  |  | |
| **C** | **الحليب ومشتقاته** | | | | | | | |
| 27 | حليب (بما في ذلك في القهوة أو مع الكورن فلايكس) | **كوب**  **أو 240 مل** |  |  |  |  |  | |
|  | نوع الحليب | € كامل الدسم € نصف دسم € خالي من الدسم | | | | | | |
| 28 | لبن (بما في ذلك في الأطباق المطبوخة) | **كوب**  **أو 240 مل** |  |  |  |  |  | |
|  | نوع اللبن | € كامل الدسم € نصف دسم € خالي من الدسم | | | | | | |
| 29 | لبنة | **50 غ**  **أو 3 ملاعق كبيرة** |  |  |  |  |  | |
|  | نوع اللّبنة | كاملة الدسم نصف دسم خالية من الدسم | | | | | | |
| 30 | أجبان <10% دهون  (ex. Picon light, Présilège, Sylphide …) | **قطعتين** |  |  |  |  |  | |
| 31 | أجبان مثل:  بلغاري بقر، شنكليش، دوبل كريم، حلوم "دايت"، موزاريل ابيضاء، بارميزان، بلدي | **30 غ أو**  **شرحتين رفيعتين** |  |  |  |  |  | |
| 32 | أجبان مثل:  بلغاري غنم، فيتا، حلوم، ريكوتا، موزاريلا صفراء، مجدولة | **30 غ أو**  **شرحتين رفيعتين** |  |  |  |  |  | |
| 33 | أجبان مثل:  بورسين، شيدار، فوندال، جبن الماعز، غرويير، قشقوان، كيري، لا فاش كيري، بيكون، سميدس | **30 غ أو**  **شرحتين رفعتين** |  |  |  |  |  | |
| 34 | قريشة | **ملعقتين كبيرتين** |  |  |  |  |  | |
| 35 | كشك | **½ كوب** |  |  |  |  |  | |
| **D** | **الفاكهة وعصير الفاكهة** | | | | | | | |
| 36 | خرما،موز، تفاح، ليمون، إجاص، دراق، نكتارين | **حبّة صغيرة** |  |  |  |  |  | |
| 37 | مشمش، خوخ، أفندي، تين، كيوي، صبيّر، بلح | **حبّتين صغيرتين** |  |  |  |  |  | |
|  | **الطعام** | **الحصة** | **عدد الحصص** | **عدد مرّات الإستهلاك** | | | | |
|  |  |  |  | **في اليوم** | **في الأسبوع** | **في الشهر** | **أبدًا** | |
| 38 | كرز، عنب، فريز، أكيدنيا | **12 حبّة** |  |  |  |  |  | |
| 39 | قشطة ، مانغا | **⅓ حبّة** |  |  |  |  |  | |
| 40 | بطيخ، شمّام | **شرحة** |  |  |  |  |  | |
| 41 | سلطة فاكهة، رمان، توت، كومبوت فاكهة | **½ كوب** |  |  |  |  |  | |
| 42 | فاكهة مجفّفة (مثل: مشمش، زبيب، تمر، تين) | **2-3 حبّة أو**  **معلقة كبيرة** |  |  |  |  |  | |
| 43 | عصير الفاكهة المعلّب | **كوب** |  |  |  |  |  | |
| 44 | عصير الفاكهة الطّازج | **كوب** |  |  |  |  |  | |
| 45 | مشروبات اصطناعية بنكهة الفاكهة (تانغ، دارينا...) | **كوب** |  |  |  |  |  | |
| **E** | **الخضار** | | | | | | | |
| 46 | بندورة، خيار، خس، ملفوف، جزر نيّ | **كوب** |  |  |  |  |  | |
| 47 | نعنع، روكا، بقلة، فجل، بصل أخضر، زعتر، فليفلة | **كوب** |  |  |  |  |  | |
| 48 | لوبية ، بامية، أرضي شوكي | **كوب** |  |  |  |  |  | |
| 49 | سبانخ، سلق، ملوخية | **كوب** |  |  |  |  |  | |
| 50 | كوسى، شمندر، جزر، قرنبيط،، بروكولي (مسلوق) | **كوب** |  |  |  |  |  | |
| 51 | قرنبيط، كوسى (مقليّ) | **2 – 3 قطع** |  |  |  |  |  | |
| 52 | فول اخضر، بازيلا خضراء | **كوب** |  |  |  |  |  | |
| 53 | باذنجان | **½ كوب** |  |  |  |  |  | |
|  | طريقة التحضير | € مشويّ € مقليّ € بالطحينة | | | | | | |
| 54 | صلصة البندورة | **½ كوب** |  |  |  |  |  | |
| 55 | فتوش، تبولة | **كوب** |  |  |  |  |  | |
| 56 | شوربة خضار | **كوب** |  |  |  |  |  | |
| 57 | مكدوس، كبيس، خضروات مخلّلة | **قطعتين** |  |  |  |  |  | |
| **F** | **اللحوم، الأسماك والبيض** | | | | | | | |
| 58 | دجاج | **90 غ** |  |  |  |  |  | |
|  | **الطعام** | **الحصة** | **عدد الحصص** | **عدد مرّات الإستهلاك** | | | | |
|  |  |  |  | **في اليوم** | **في الأسبوع** | **في الشهر** | **أبدًا** | |
|  | كيف تتناولين الدجاج عادةً؟ | € سفينة دجاج بدون جلدة مقليّة € سفينة دجاج بدون جلدة مشويّة  € سفينة دجاج مع جلدة مقليّة € سفينة دجاج مع جلدة مشويّة  € فخذ دجاج بدون جلدة مقليّ € فخذ دجاج بدون جلدة مشويّ  € فخذ دجاج مع جلدة مقليّ € فخذ دجاج مع جلدة مشويّ  € اسكالوب دجاج € اسكالوب دجاج مع جبنة وجانبون | | | | | | |
| 59 | ناغتزNuggets | **6 قطع** |  |  |  |  |  | |
| 60 | لحم بقر (ستيك 6 قطع) | **90 غ** |  |  |  |  |  | |
|  | نوع اللحمة | € مدهنة € هبرة | | | | | | |
| 61 | لحم غنم | **90 غ** |  |  |  |  |  | |
|  | نوع اللحمة | € مدهنة € هبرة | | | | | | |
| 62 | بيض | **بيضة واحدة** |  |  |  |  |  | |
|  |  | € البياض فقط € الصفار فقط € مقليّ € مسلوق | | | | | | |
| 63 | تونة | **90 غ** |  |  |  |  |  | |
|  | نوع التونة | معلّبة بالماء € معلّبة بالزيت | | | | | | |
| 64 | سردين معلب | **3 قطع**  **90 غ** |  |  |  |  |  | |
| 65 | سمك (غير التونة) مقلي | **90 غ** |  |  |  |  |  | |
| 66 | سمك (غير التونة) مشوي | **90 غ** |  |  |  |  |  | |
| 67 | ثمارالبحر: بلح البحرles)umo)، أو القشريات مثل سلطعون (rabec)، سوريمي، قريدس... | **30 غ** |  |  |  |  |  | |
| 68 | جامبون | **شرحتين**  **أو 30 غ** |  |  |  |  |  | |
| 69 | جامبون حبش | **شرحتين**  **أو 30 غ** |  |  |  |  |  | |
| 70 | شاركوتري (غيرالجامبون): مثل سلامي، مورتديلا...) | **شرحتين**  **أو 30 غ** |  |  |  |  |  | |
| 71 | قصبة، قلوب وأكباد | **30 غ** |  |  |  |  |  | |
| 72 | شاورما (لحمة أو دجاج) | **90 غ** |  |  |  |  |  | |
| 73 | هوت دوج | **قطعة** |  |  |  |  |  | |
| 74 | برغر | **قطعة** |  |  |  |  |  | |
|  | نوع البرغر | € لحمة (بقر) € دجاج € سمك € غير ذلك: .................... | | | | | | |
| 75 | عصافير | **حبتين** |  |  |  |  |  | |
|  | طريقة التحضير | مقليّة € مشويّة | | | | | | |
|  | **الطعام** | **الحصة** | **عدد الحصص** | **عدد مرّات الإستهلاك** | | | | |
|  |  |  |  | **في اليوم** | **في الأسبوع** | **في الشهر** | **أبدًا** | |
| 76 | ضفادع مقلية | **حبتين** |  |  |  |  |  | |
| 77 | قاورما | **معلقتين كبيرتين** |  |  |  |  |  | |
| 78 | نقانق | **2 – 3 قطع** |  |  |  |  |  | |
| 79 | بسترما | **شرحة** |  |  |  |  |  | |
| 80 | سجق | **قطعة** |  |  |  |  |  | |
| 81 | بايكون (Bacon) | **شرحة**  **أو 30 غ** |  |  |  |  |  | |
| 82 | سوشي | **قطعة** |  |  |  |  |  | |
| **G** | **توابل، مكسّرات وقلوبات** | | | | | | | |
| 83 | مايونيز عادي | **ملعقة صغيرة** |  |  |  |  |  | |
| 84 | مايونيز دايت | **ملعقة كبيرة** |  |  |  |  |  | |
| 85 | كاتشب | **ملعقة كبيرة** |  |  |  |  |  | |
| 86 | صلصة الصويا | **ملعقة كبيرة** |  |  |  |  |  | |
|  | نوع الصلصة | € عاديّة € قليلة الملح | | | | | | |
| 87 | مكعّب مرق | **مكعّب** |  |  |  |  |  | |
| 88 | كاجو، لوز | **6 حبّات** |  |  |  |  |  | |
|  | النوع | € نيّ € محمّص غير مملّح € محمّص و مملّح | | | | | | |
| 89 | فستق | **6 حبّة** |  |  |  |  |  | |
|  | النوع | € نيّ € محمّص غير مملّح € محمّص و مملّح | | | | | | |
| 90 | جوز | **حبتين** |  |  |  |  |  | |
| 91 | صنوبر | **ملعقة كبيرة** |  |  |  |  |  | |
| 92 | بندق | **8 حبّات** |  |  |  |  |  | |
| 93 | زبدة الفول السوداني | **ملعقة كبيرة** |  |  |  |  |  | |
| 94 | طحينة (بما في ذلك في الأطباق المطبوخة) | **ملعقتين صغيرتين** |  |  |  |  |  | |
| 95 | أفوكادو | **شرحة** |  |  |  |  |  | |
| 96 | زيتون | **5 – 8 حبات** |  |  |  |  |  | |
| 97 | جوز الهند | **ملعقتين كبيرتين** |  |  |  |  |  | |
| 98 | بزر اليقطين أو غيرها | **ملعقة كبيرة** |  |  |  |  |  | |
|  | **الطعام** | **الحصة** | **عدد الحصص** | **عدد مرّات الإستهلاك** | | | | |
|  |  |  |  | **في اليوم** | **في الأسبوع** | **في الشهر** | **أبدًا** | |
| **H** | **السكاكر والحلويات** | | | | | | | |
| 99 | سكر، عسل، مربى، دبس، حبة بانبون | **ملعقة كبيرة** |  |  |  |  |  | |
| 100 | شوكولا المرح (مثل: نوتيلا) | **ملعقة صغيرة** |  |  |  |  |  | |
| 101 | شوكولا بالحليب، شوكولا مرّ | **10 غ**  **أو مربع شوكولا** |  |  |  |  |  | |
| 102 | شوكولا لوح | **حسب الماركة** |  |  |  |  |  | |
|  | حددي الماركة: |  | | | | | | |
| 103 | شوكولا بالوايفر | **حسب الماركة** |  |  |  |  |  | |
|  | حددي الماركة: |  | | | | | | |
| 104 | حلاوة | **ملعقة كبيرة** |  |  |  |  |  | |
| 105 | بسكويت دون كريمة | **قطعة متوسطة** |  |  |  |  |  | |
| 106 | بسكويت مع كريمة | **قطعة متوسطة** |  |  |  |  |  | |
| 107 | كيك ناشف | **قطعة صغيرة** |  |  |  |  |  | |
| 108 | كيك بالكريمة | **قطعة صغيرة** |  |  |  |  |  | |
| 109 | كريب، غوفر، بان كيك | **30 غ**  **أو قطعة** |  |  |  |  |  | |
| 110 | بوظة على حليب | **1 سكوب**  **أو ½ كوب** |  |  |  |  |  | |
| 111 | بوظة على ثلج | **ستيك** |  |  |  |  |  | |
| 112 | بوظة سوربيه | **1 سكوب**  **أو ½ كوب** |  |  |  |  |  | |
| 113 | بوظة على لبن | **1 سكوب**  **أو ½ كوب** |  |  |  |  |  | |
| 114 | حلويات عربية (مثل: بقلاوة، معمول...) | **قطعة** |  |  |  |  |  | |
| 115 | حلويات عربية (مثل زنود الست، حلاوة الجبن...) | **قطعة** |  |  |  |  |  | |
| 116 | كنافة | **قطعة** |  |  |  |  |  | |
| 117 | أرز بالحليب، مهلبية، كاسترد | **كاسة** |  |  |  |  |  | |
| 118 | مغلي | **كاسة** |  |  |  |  |  | |
| 119 | جلو | **كاسة** |  |  |  |  |  | |
|  |  | € عادي € دايت | | | | | | |
| 120 | اكلير، تارت بالفاكهة، ميل فاي | **قطعة** |  |  |  |  |  | |
| 121 | بيتي فور | **قطعة** |  |  |  |  |  | |
|  | **الطعام** | **الحصة** | **عدد الحصص** | **عدد مرّات الإستهلاك** | | | | |
|  |  |  |  | **في اليوم** | **في الأسبوع** | **في الشهر** | **أبدًا** | |
| **I** | **المعجّنات** | | | | | | | |
| 122 | بيتزا | **قطعة** |  |  |  |  |  | |
|  |  | € بوشيه € مثلّثة | | | | | | |
| 123 | مناقيش | **منقوشة حجم وسط** |  |  |  |  |  | |
|  |  | € زعتر € جبنة € كشك | | | | | | |
| 124 | فطائر سبانخ | **قطعتين** |  |  |  |  |  | |
| 125 | فطائر جبنة و رقاقات | **قطعتين** |  |  |  |  |  | |
| 126 | لحم بالعجين | **قطعة** |  |  |  |  |  | |
|  |  | € بوشيه € حجم وسط | | | | | | |
| 127 | كرواسان سادة، كرواسان زعتر | **قطعة** |  |  |  |  |  | |
| 128 | كرواسان جبنة | **قطعة** |  |  |  |  |  | |
| 129 | كرواسان شوكولا | **قطعة** |  |  |  |  |  | |
| 130 | خبز بالشوكولا، بان أوليه شوكولا | **قطعة** |  |  |  |  |  | |
| 131 | دونتس | **قطعة** |  |  |  |  |  | |
| 132 | بريوش | **قطعة صغيرة** |  |  |  |  |  | |
| **J** | **المأكولت المالحة** | | | | | | | |
| 133 | شيبس | **30 غ**  **أو 10 حبّات** |  |  |  |  |  | |
| 134 | بوشار (قليل الزيت) | **3 أكواب** |  |  |  |  |  | |
| 135 | بسكويت مملّح | **كيس** |  |  |  |  |  | |
| **K** | **صلصات، زيوت ودهون** | | | | | | | |
| 136 | زيت الزيتون | **ملعقة صغيرة** |  |  |  |  |  | |
| 137 | زيت دوّار الشمس | **ملعقة صغيرة** |  |  |  |  |  | |
| 138 | زيت الكانولا | **ملعقة صغيرة** |  |  |  |  |  | |
| 139 | زيت الذرة | **ملعقة صغيرة** |  |  |  |  |  |  |
| 140 | زيت جوز الهند | **ملعقة صغيرة** |  |  |  |  |  |  |
| 141 | زيت النخيل | **ملعقة صغيرة** |  |  |  |  |  |  |
| 142 | زبدة عاديّة | **ملعقة صغيرة** |  |  |  |  |  |  |
| 143 | سمنة نباتيّة | **ملعقة صغيرة** |  |  |  |  |  |  |
| 144 | سمنة حيوانيّة | **ملعقة صغيرة** |  |  |  |  |  | |

|  | **الطعام** | **الحصة** | **عدد الحصص** | **عدد مرّات الإستهلاك** | | | | |
| --- | --- | --- | --- | --- | --- | --- | --- | --- |
|  |  |  |  | **في اليوم** | **في الأسبوع** | **في الشهر** | **أبدًا** | |
| **L** | **مشروبات** | | | | | | | |
| 145 | مياه معدنيّة | **كوب** |  |  |  |  |  | |
| 146 | شاي | **فنجان كبير** |  |  |  |  |  | |
| 147 | زهورات | **فنجان كبير** |  |  |  |  |  | |
| 148 | قهوة | **فنجان صغير** |  |  |  |  |  | |
| 149 | نسكافيه مع كوفي مايت | **فنجان كبير** |  |  |  |  |  | |
| 150 | نسكافيه دون كوفي مايت | **فنجان كبير** |  |  |  |  |  | |
| 151 | نسكافيه ديكاف مع كوفي مايت | **فنجان كبير** |  |  |  |  |  | |
| 152 | نسكافيه ديكاف دون كوفي مايت | **فنجان كبير** |  |  |  |  |  | |
| 153 | شاي مثلّج (ايس تي) | **كوب** |  |  |  |  |  |  |
| 154 | مشروبات غازيّة (بيبسي..) | **كوب** |  |  |  |  |  |  |
| 155 | مشروبات غازيّة دايت | **كوب** |  |  |  |  |  |  |
| 156 | مشروبات الطاقة (ريد بل..) | **كوب** |  |  |  |  |  |  |
| 157 | مشروبات روحيّة (كحول) | **كوب** |  |  |  |  |  |  |
