# Supplementary material for: Reproducibility and relative validity of a food frequency questionnaire to assess dietary intake of adults living in a Mediterranean country
Source: PLoS One. 2019 Jun 17;14(6):e0218541. doi: 10.1371/journal.pone.0218541 (PMC6576765; doi:10.1371/journal.pone.0218541)
Supplement: S2 File — (DOCX) [file pone.0218541.s002.docx]

| **Food Frequency Questionnaire for Mediterranean Food** | | | | | | | | |
| --- | --- | --- | --- | --- | --- | --- | --- | --- |
|  | **Food** | **Portion** | | **Number of portions** | **Number of consumptions** | | | |
|  |  |  |  |  | **In a day** | **In a week** | **In a month** | **Never** |
| **A** | **Bread and cereals** |  | |  |  |  |  |  |
| 1 | Arabic bread (including sandwiches) | ¼ large loaf  ½ medium loaf | |  |  |  |  |  |
| 2 | “Markouk” bread / “Tannour” bread | ½ markouk  1 tannour | |  |  |  |  |  |
| 3 | Burger bread | 1 piece | |  |  |  |  |  |
| 4 | French baguette | 30 g or 1 mini-baguette | |  |  |  |  |  |
| 5 | Pain au lait | 1 piece | |  |  |  |  |  |
| 6 | Toast, cracotte | 3 pieces | |  |  |  |  |  |
| 7 | Pain de mie | 2 pieces | |  |  |  |  |  |
| 8 | Lebanese Kaake | 8 round pieces  3 medium fingers  1 large finger | |  |  |  |  |  |
|  | Specify the kind of food | ( ) White ( ) Brown(whole wheat) ( ) Other: | | | | | | |
| 9 | Corn flakes normal | ½ cup | |  |  |  |  |  |
| 10 | Corn flakes rich in fibers | ¾ cup | |  |  |  |  |  |
| 11 | Cereal bar | 30 g or 1 piece | |  |  |  |  |  |
| 12 | Oat | ½ cup | |  |  |  |  |  |
| **B** | **Potato, pasta, rice and legumes** | | | | | | | |
| 13 | Rice, cooked | 1 cup (15 tbsp) | |  |  |  |  |  |
| 14 | Lebanese stuffed dishes | 12 wine leaves  6 cabbage leaves  3 zucchinis | |  |  |  |  |  |
| 15 | Cooked bulgur, cooked freekeh, moghrabieh | 1 cup (10 tbsp) | |  |  |  |  |  |
| 16 | Kibbeh | 1 medium slice or 2 mini kibbeh pieces | |  |  |  |  |  |
| 17 | Pasta, cooked | 1 cup | |  |  |  |  |  |
| 18 | Potato (smashed or puree) | 1 medium piece or 1 cup | |  |  |  |  |  |
| 19 | Potato, fries | 10 to 12 pieces | |  |  |  |  |  |
| 20 | Chestnut | 4 to 5 pieces | |  |  |  |  |  |
| 21 | Quinoa, cooked | 5 tbsp | |  |  |  |  |  |
| 22 | Green peas | ½ cup or 5 tbsp | |  |  |  |  |  |
| 23 | Corn | ½ cup or 5 tbsp | |  |  |  |  |  |
| 24 | Fava beans, kidney beans | 1 cup or 10 tbsp | |  |  |  |  |  |
|  | **Food** | **Portion** | | **Number of portions** | **Number of consumptions** | | | |
|  |  |  |  |  | **In a day** | **In a week** | **In a month** | **Never** |
| 25 | Lentils | 1 cup or 10 tbsp | |  |  |  |  |  |
| 26 | Chickpeas | 1 cup or 10 tbsp | |  |  |  |  |  |
| **C** | **Milk and dairy products** | | | | | | | |
| 27 | Milk (including in coffee or corn flakes) | 1 cup or 240 ml | |  |  |  |  |  |
|  | Type of milk | ( ) Full fat ( ) Reduced-fat milk ( ) Skimmed milk | | | | | | |
| 28 | Yogurt (including in cooked dishes) | 1 cup or 240 ml | |  |  |  |  |  |
|  | Type of yogurt | ( ) Full fat ( ) Reduced-fat milk ( ) Skimmed milk | | | | | | |
| 29 | Labneh | 50 g or 3 tbsp | |  |  |  |  |  |
|  | Type of yogurt | ( ) Full fat ( ) Reduced-fat milk ( ) Skimmed milk | | | | | | |
| 30 | Cheese >10% fat  (ex. Picon light, Présilège, Sylphide …) | 2 pieces | |  |  |  |  |  |
| 31 | Cheese such as:  Bulghari cow, Shanklish, Double creme, Haloum light, Mozarella, Parmesan | 30 g or 2 thin slices | |  |  |  |  |  |
| 32 | Cheese such as:  Bulghari sheep, Haloum, Ricotta, yellow mozzarella | 30 g or 2 thin slices | |  |  |  |  |  |
| 33 | Cheese such as: Boursin, Cheddar, Fondel, Goat Cheese, Gruyere, Kashkawan, La vache kiri, Smedes, Picon | 30 g or 2 thin slices | |  |  |  |  |  |
| 34 | Kariche | 2 tbsp | |  |  |  |  |  |
| 35 | Kechek | ½ cup | |  |  |  |  |  |
| **D** | **Fruits and fruit juices** | | | | | | | |
| 36 | Banana, apple, lemon, pear, peach, nectarine | 1 small piece | |  |  |  |  |  |
| 37 | Apricot, Peach, clementine, figs, Kiwi, dates | 1 small piece | |  |  |  |  |  |
| 38 | Cherry, Grape, Strawberries | 12 pieces | |  |  |  |  |  |
| 39 | Mango, cherimoya | 1/3 piece | |  |  |  |  |  |
| 40 | Watermelon, melon | 1 slice | |  |  |  |  |  |
| 41 | Fruit salad or compote, pomegranate, berries | ½ cup | |  |  |  |  |  |
|  | **Food** | **Portion** | | **Number of portions** | **Number of consumptions** | | | |
|  |  |  |  |  | **In a day** | **In a week** | **In a month** | **Never** |
| 42 | Dried fruits (such as: apricots, raisins, dates, figs) | 2-3 pieces or 1 tbsp | |  |  |  |  |  |
| 43 | Commercial fruit juice | 1 cup | |  |  |  |  |  |
| 44 | Fresh fruit juice | 1 cup | |  |  |  |  |  |
| 45 | Artificially flavored juices (Tang, Darina…) | 1 cup | |  |  |  |  |  |
| **E** | **Vegetables** | | | | | | | |
| 46 | Tomato, cucumber, lettuce, cabbage | 1 cup | |  |  |  |  |  |
| 47 | Mint, rocca, basil, radish, green onion, thyme, pepper | 1 cup | |  |  |  |  |  |
| 48 | Okra, artichoke, green beans | 1 cup | |  |  |  |  |  |
| 49 | Spinach, chard, mloukhiye | 1 cup | |  |  |  |  |  |
| 50 | Zucchini, beetroot, carrots, cauliflower, broccoli (boiled) | 1 cup | |  |  |  |  |  |
| 51 | Zucchini, cauliflower (fried) | 2 to 3 pieces | |  |  |  |  |  |
| 52 | Green fava beans, green peas | 1 cup | |  |  |  |  |  |
| 53 | Eggplant | ½ cup | |  |  |  |  |  |
|  | Preparation method | ( ) Grilled ( ) Fried ( ) With tahini | | | | | | |
| 54 | Tomato sauce | ½ cup | |  |  |  |  |  |
| 55 | Fattouch, tabbouleh | 1 cup | |  |  |  |  |  |
| 56 | Vegetables soup | 1 cup | |  |  |  |  |  |
| 57 | Makdous, pickeled vegetables | 2 pieces | |  |  |  |  |  |
| **F** | **Meats, fish and eggs** | | | | | | | |
| 58 | Chicken | 90 g | |  |  |  |  |  |
|  | How do you usually eat chicken? | ( ) Breast without skin, fried ( ) Breast without skin, grilled  ( ) Breast with skin, fried ( ) Breast with skin, grilled  ( ) Thigh without skin, fried ( ) Thigh without skin, grilled  ( ) Thigh with skin, fried ( ) Thigh with skin, grilled  ( ) Escalope ( ) Escalope with ham and cheese | | | | | | |
| 59 | Chicken nuggets | 6 pieces | |  |  |  |  |  |
| 60 | Meat, beef | 90 g | |  |  |  |  |  |
|  | Type of meat | ( ) Fatty ( ) Lean | | | | | | |
| 61 | Meat, sheep | 90 g | |  |  |  |  |  |
|  | Type of meat | ( ) Fatty ( ) Lean | | | | | | |
|  | **Food** | **Portion** | **Number of portions** | | **Number of consumptions** | | | |
|  |  |  |  |  | **In a day** | **In a week** | **In a month** | **Never** |
| 62 | Eggs | 1 egg |  | |  |  |  |  |
|  |  | ( ) Only white ( ) Only yellow ( ) Fried ( ) Boiled | | | | | | |
| 63 | Tuna | 90 g |  | |  |  |  |  |
|  |  | ( ) Canned in water ( ) Canned in oil | | | | | | |
| 64 | Sardine, canned | 90 g or 3 pieces |  | |  |  |  |  |
| 65 | Fish, fried | 90 g |  | |  |  |  |  |
| 66 | Fish, grilled | 90 g |  | |  |  |  |  |
| 67 | Seafood | 30 g |  | |  |  |  |  |
| 68 | Ham | 30 g or 2 pieces |  | |  |  |  |  |
| 69 | Turkey ham | 30 g or 2 pieces |  | |  |  |  |  |
| 70 | Charcuterie (other than ham): salami etc… | 30 g or 2 pieces |  | |  |  |  |  |
| 71 | Liver, other organs | 30 g |  | |  |  |  |  |
| 72 | Shawarma (meat or chicken) | 90 g |  | |  |  |  |  |
| 73 | Hotdog | 1 piece |  | |  |  |  |  |
| 74 | Burger | 1 piece |  | |  |  |  |  |
|  | Type of burger: | ( ) meat(beef) ( ) chicken ( ) fish | | | | | | |
| 75 | Birds | 2 pieces |  | |  |  |  |  |
|  | Preparation method | ( ) grilled ( ) fried | | | | | | |
| 76 | Frogs, fried | 2 pieces |  | |  |  |  |  |
| 77 | Kawarma | 2 tbsp |  | |  |  |  |  |
| 78 | Makanek (sausages) | 2 pieces |  | |  |  |  |  |
| 79 | Basterma | 1 slice |  | |  |  |  |  |
| 80 | Sojok(Armenian sausage) | 1 piece |  | |  |  |  |  |
| 81 | Bacon | 1 slice or 30 g |  | |  |  |  |  |
| 82 | Sushi | 1 piece |  | |  |  |  |  |
| G | Seasoning and nuts | | | | | | | |
| 83 | Mayonnaise, normal | 1 tsp |  | |  |  |  |  |
| 84 | Mayonnaise, light | 1 tbsp |  | |  |  |  |  |
| 85 | Ketchup | 1 tbsp |  | |  |  |  |  |
| 86 | Soya sauce | 1 tbsp |  | |  |  |  |  |
|  | Type of sauce: | ( ) Normal ( ) Low in salt | | | | | | |
| 87 | Bouillon cube | 1 cube |  | |  |  |  |  |
| 88 | Almonds, cashew nuts | 6 pieces |  | |  |  |  |  |
|  | Type of nuts: | ( ) raw ( ) grilled ( ) grilled and salted | | | | | | |
| 89 | Pistachio | 6 pieces |  | |  |  |  |  |
|  | Type of nuts: | ( ) raw ( ) grilled ( ) grilled and salted | | | | | | |
| 90 | Walnuts | 2 pieces |  | |  |  |  |  |
| 91 | Pine nut | 1 tbsp |  | |  |  |  |  |
| 92 | Hazelnut | 8 pieces |  | |  |  |  |  |
|  | **Food** | **Portion** | **Number of portions** | | **Number of consumptions** | | | |
|  |  |  |  |  | **In a day** | **In a week** | **In a month** | **Never** |
| 93 | Peanut butter | 1 tbsp |  | |  |  |  |  |
| 94 | Tahini | 2 tsp |  | |  |  |  |  |
| 95 | Avocado | 1 slice |  | |  |  |  |  |
| 96 | Olives | 5 to 8 pieces |  | |  |  |  |  |
| 97 | Coconut | 2 tbsp |  | |  |  |  |  |
| 98 | Pumpkin seeds | 1 tbsp |  | |  |  |  |  |
| **H** | **Sugars, sweets** |  | | | | | | |
| 99 | Sugar, honey, jam, molasses, candie | 1 tbsp |  | |  |  |  |  |
| 100 | Chocolate spread (nutella) | 1 tsp |  | |  |  |  |  |
| 101 | Chocolate milk or dark | 10 g or 1 square of chocolate |  | |  |  |  |  |
| 102 | Chocolate bar | Depending on the brand |  | |  |  |  |  |
|  | Specify the brand: |  | | | | | | |
| 103 | Chocolate wafer | Depending on the brand |  | |  |  |  |  |
|  | Specify the brand: |  |  | |  |  |  |  |
| 104 | Halawa | 1 tbsp |  | |  |  |  |  |
| 105 | Biscuit without cream | 1 medium piece |  | |  |  |  |  |
| 106 | Biscuit with cream | 1 medium piece |  | |  |  |  |  |
| 107 | Sponge cake | Small piece |  | |  |  |  |  |
| 108 | Cake with cream | Small piece |  | |  |  |  |  |
| 109 | Pan cake, crepe, gauffre | 30 g or 1 piece |  | |  |  |  |  |
| 110 | Ice cream with milk | 1 scoop or ½ cup |  | |  |  |  |  |
| 111 | Ice cream stick | 1 stick |  | |  |  |  |  |
| 112 | Ice cream sorbet | 1 scoop or ½ cup |  | |  |  |  |  |
| 113 | Frozen yogurt | 1 scoop or ½ cup |  | |  |  |  |  |
| 114 | Arabic sweets (baklawa, maamoul….) | 1 piece |  | |  |  |  |  |
|  | **Food** | **Portion** | **Number of portions** | | **Number of consumptions** | | | |
|  |  |  |  |  | **In a day** | **In a week** | **In a month** | **Never** |
| 115 | Arabic sweets (znoud el sit, halawat el jibn...) | 1 piece |  | |  |  |  |  |
| 116 | Kneffeh | 1 piece |  | |  |  |  |  |
| 117 | Custard, rice with milk | 1 cup |  | |  |  |  |  |
| 118 | Meghlé | 1 cup |  | |  |  |  |  |
| 119 | Jello | 1 cup |  | |  |  |  |  |
|  |  | ( ) Normal ( ) diet | | | | | | |
| 120 | Éclair, fruit tart, mille-feuille | 1 piece |  | |  |  |  |  |
| 121 | Petit four | 1 piece |  | |  |  |  |  |
| **I** | **Pastries sweet or salty** | | | | | | | |
| 122 | Pizza | 1 slice |  | |  |  |  |  |
|  |  | ( ) triangle ( ) mini pizza | | | | | | |
| 123 | Manaeech | Medium man’ouche |  | |  |  |  |  |
|  |  | ( ) thyme ( ) cheese ( ) kecheck | | | | | | |
| 124 | Spinach pie | 2 pieces |  | |  |  |  |  |
| 125 | Cheese pie or rolls | 2 pieces |  | |  |  |  |  |
| 126 | Meat pie | 1 slice |  | |  |  |  |  |
|  |  | ( ) mini size ( ) medium | | | | | | |
| 127 | Croissant plain, croissant thyme | 1 piece |  | |  |  |  |  |
| 128 | Croissant, cheese | 1 piece |  | |  |  |  |  |
| 129 | Croissant, chocolat | 1 piece |  | |  |  |  |  |
| 130 | Chocolate bread | 1 piece |  | |  |  |  |  |
| 131 | Donuts | 1 piece |  | |  |  |  |  |
| 132 | Brioche | 1 piece |  | |  |  |  |  |
| **J** | **Salty snacks** |  |  | |  |  |  |  |
| 133 | Chips | 30 g or 10 pieces |  | |  |  |  |  |
| 134 | Pop corn | 3 cups |  | |  |  |  |  |
| 135 | Salty biscuits | 1 small bag |  | |  |  |  |  |
| **K** | **Dressings, oils and fats** | | | | | | | |
| 136 | Olive oil | 1 tsp |  | |  |  |  |  |
| 137 | Sunflower oil | 1 tsp |  | |  |  |  |  |
| 138 | Canola oil | 1 tsp |  | |  |  |  |  |
| 139 | Corn oil | 1 tsp |  | |  |  |  |  |
| 140 | Coconut oil | 1 tsp |  | |  |  |  |  |
| 141 | Palm oil | 1 tsp |  | |  |  |  |  |
| 142 | Butter | 1 tsp |  | |  |  |  |  |
| 143 | Vegetable margarine | 1 tsp |  | |  |  |  |  |
| 144 | Animal margarine | 1 tsp |  | |  |  |  |  |
|  | **Food** | **Portion** | **Number of portions** | | **Number of consumptions** | | | |
|  |  |  |  |  | **In a day** | **In a week** | **In a month** | **Never** |
| **L** | **Beverages** | | | | | | | |
| 145 | Mineral water | 1 cup |  | |  |  |  |  |
| 146 | Tea | 1 big cup |  | |  |  |  |  |
| 147 | Infusions | 1 big cip |  | |  |  |  |  |
| 148 | Coffee | 1 small cup |  | |  |  |  |  |
| 149 | Nescafe with coffee mate | 1 big cup |  | |  |  |  |  |
| 150 | Nescafe without coffee mate | 1 big cup |  | |  |  |  |  |
| 151 | Nescafe decaffeinated with coffee mate | 1 big cup |  | |  |  |  |  |
| 152 | Nescafe decaffeinated without coffee mate | 1 big cup |  | |  |  |  |  |
| 153 | Iced tea | 1 cup |  | |  |  |  |  |
| 154 | Carbonated beverages | 1 cup |  | |  |  |  |  |
| 155 | Carbonated beverages diet | 1 cup |  | |  |  |  |  |
| 156 | Energy drinks (RedBull…) | 1 cup |  | |  |  |  |  |
| 157 | Spirits and alcohol | 1 cup |  | |  |  |  |  |
